# Vehicle Detection and Tracking Using Thermal Cameras in Adverse Visibility Conditions

**DOI:** 10.3390/s22124567

**Published:** 2022-06-17

**Authors:** Abhay Singh Bhadoriya, Vamsi Vegamoor, Sivakumar Rathinam

**Affiliations:** 1 Department of Mechanical Engineering, Texas A&M University, College Station, TX 77843, USA; abhay.singh@tamu.edu (A.S.B.); vamsikrishna95@tamu.edu (V.V.); 2 Goodyear Tire and Rubber Company, Akron, OH 44306, USA

**Keywords:** object tracking, sensor fusion, thermal cameras

## Abstract

Level 5 autonomy, as defined by the Society of Automotive Engineers, requires the vehicle to function under all weather and visibility conditions. This sensing problem becomes significantly challenging in weather conditions that include events such as sudden changes in lighting, smoke, fog, snow, and rain. No standalone sensor currently in the market can reliably perceive the environment in all conditions. While regular cameras, lidars, and radars will suffice for typical driving conditions, they may fail in some edge cases. The goal of this paper is to demonstrate that the addition of Long Wave Infrared (LWIR)/thermal cameras to the sensor stack on a self-driving vehicle can help fill this sensory gap during adverse visibility conditions. In this paper, we trained a machine learning-based image detector on thermal image data and used it for vehicle detection. For vehicle tracking, Joint Probabilistic Data association and Multiple Hypothesis Tracking approaches were explored where the thermal camera information was fused with a front-facing radar. The algorithms were implemented using FLIR thermal cameras on a 2017 Lincoln MKZ operating in College Station, TX, USA. The performance of the tracking algorithm has also been validated in simulations using Unreal Engine.

## 1. Introduction

It is well known that over 90% of traffic accidents on roads are caused by human error [1,2]. The primary goal of fully autonomous driving is to eliminate such accidents by removing the human driver from the equation. For such vehicles to operate safely, they need to be able to perceive other road users (cars, bikes, pedestrians, etc.) as well as, if not better than, a human driver. Currently, no single sensor exists on the market that can perform this task reliably under all conditions. Instead, autonomous driving systems utilize a combination of sensors for environment perception.

Radars, first developed for military and aviation applications, are used for measuring the distance and speed of objects. They have been used by automotive manufacturers in the last couple of decades for implementing adaptive cruise control and collision avoidance/emergency braking features [3]. Lidars, or laser scanners, offer better angular resolution and, consequently, enable better object classification, especially when combined with neural network-based approaches [4,5]. Cameras yield the highest resolution and are usually designed to work in the visible range (400 to 700 nanometers). Digital (or ‘RGB’) cameras typically output color information in three channels (Red, Green, and Blue) for each pixel and are best suited for object classification.

A fully autonomous vehicle, as defined by the Society of Automotive Engineers, must be able to perform all driving functions (including perception) under all conditions [6]. In order to achieve Level 5 autonomy, we need to design systems that work reliably even in adverse weather and visibility conditions, without the need for human intervention. Radars, lidars, and RGB cameras have different operating characteristics in different situations.

**Figure 1:**
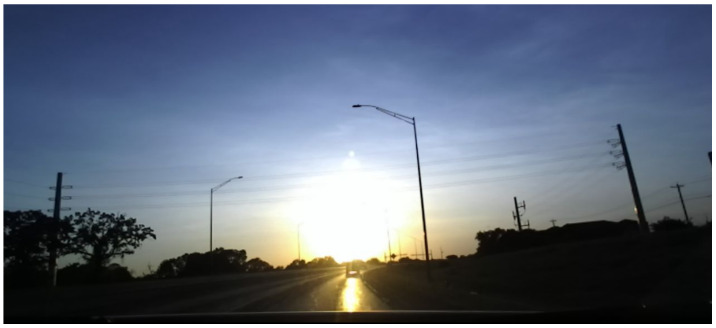
RGB camera hindered by direct sunlight, taken with a ZED camera from StereoLabs at 1080p resolution. Reprinted with permission from earlier conference paper Ref. [11]. ©2021, SAE International.

The primary drawback of automotive radars is the low spatial resolution and, consequently, their inability to distinguish the type of obstacle (‘object classification’) from measurements. It has been demonstrated that Lidar sensors perform poorly in heavy rain or fog due to reflections from the water droplets [7,8] (though some of it can be mitigated using better signal processing and different wavelengths). RGB cameras yield limited information at night due to low lighting. They can only sense the area covered by the headlights and are further hindered by headlight blooms from oncoming vehicles. Moreover, they are severely disrupted by direct glare while driving directly in the direction of sunrise or sunset, as shown in Figure 1.

Loss of visibility under intense sunlight can be fatal, as demonstrated by the failure of Tesla’s AutoPilot system to detect the side of a trailer in 2016 [9]. Recently, ref. [10] presented an approach for improving visibility in direct sunlight by using a High Dynamic Range (HDR) camera that captures multiple frames at different exposure settings to create a composite image. Apart from adding additional computational expenses, the same approach is not guaranteed to work for night-time autonomous driving and may not allow high frame rates.

**Figure 2:**
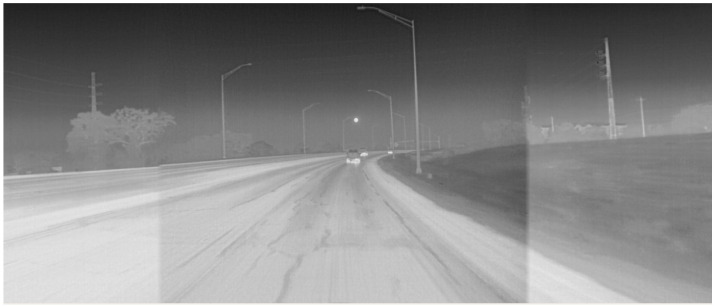
Thermal cameras are resilient to direct sunlight. Showing the same scene as in Figure 1. Reprinted with permission from earlier conference paper Ref. [11]. ©2021, SAE International.

Thermal cameras, especially those operating over the Long Wavelength Infrared (LWIR) spectrum (8000 to 14,000 nanometers), are inherently resilient to direct sunlight, as demonstrated in Figure 2, which presents the same scene as in Figure 1, but as seen from a thermal camera. Thermal/infrared cameras have been successfully used for military and civilian applications owing to their robustness to poor visibility conditions. For example, ref. [12] demonstrates techniques to identify people in challenging visibility conditions, including in rain. Moreover, a study by [13] demonstrated the superior performance of LWIR cameras over lidars and RGB cameras in foggy conditions for identifying pedestrians. More recently, auto manufacturers have started to implement thermal cameras as night vision systems as part of their driver assistance features [14]. Given their robustness to different weather conditions, we believe that thermal cameras have the potential to play an important role in autonomous driving in the near future.

There has been a rise in research interest involving thermal cameras for automotive applications. Kristoffersen et al. [15] were able to achieve good performance in the task of counting pedestrians using stereo thermal cameras in spite of the pedestrians occluding each other. There have also been studies that combine thermal camera information with lidar data for self-driving [16,17], but they have been more focused on the calibration aspect rather than data association/sensor fusion. Consequently, in this paper, we propose an object detection and tracking system that utilizes thermal cameras in combination with an automotive radar to complete the perception task in adverse visibility conditions. With this, we demonstrate that the edge cases that are challenging to the more commonly used sensors can be covered by thermal cameras.

In Section 2, we present the details of the sensor hardware used and the data acquisition. Section 3 describes the data association algorithms used for processing measurements from the cameras and radar. Section 4 summarizes the simulation-based validation work that was performed using Unreal Engine, followed by a conclusion in Section 5.

Portions of this journal paper have been previously published as a conference paper [11]. This work completes [11] by including validation work that was performed since the publication of the conference paper. We have also since uploaded a dataset [18] containing sensor data from lidars, five thermal cameras, a radar, IMU, and vehicle CAN bus (https://doi.org/10.15787/VTT1/B3VKEA (accessed on 14 June 2022)).

## 2. Sensor Configuration and Data Acquisition

### 2.1. Hardware

**Figure 3:**
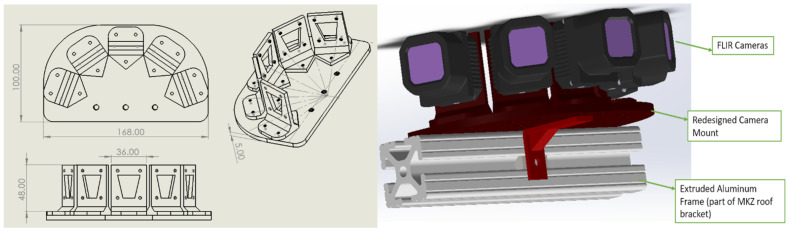
Mount designed for the five thermal cameras.

For radar, we used a Delphi/Aptiv ESR 2.5, which was mounted to the front bumper of our test vehicle (a 2017 Lincoln MKZ). For thermal vision, we used five FLIR Automotive Development Kit cameras, each with a 50-degree field-of-view (FOV). Since each individual camera has a relatively narrow field of view, a camera mount was designed with the five cameras arranged radially to create a panorama with a FOV of 190 degrees. The main reason for using five cameras is to allow for consistent tracking of vehicles and pedestrians over a longer duration of time. If only a single camera with a 50-degree field of view were to be used, then only one or two lanes of traffic could be tracked. With the wide 190-degree field of view, we could track the complete view in front of the vehicle and also cross-traffic (pedestrians crossing in front, vehicles at intersections, etc.). This is also an aspect that sets this work apart from the majority of the other studies involving thermal camera systems in this area. Perspective drawings of the mount and a 3D render of the mount are presented in Figure 3.

**Figure 4:**
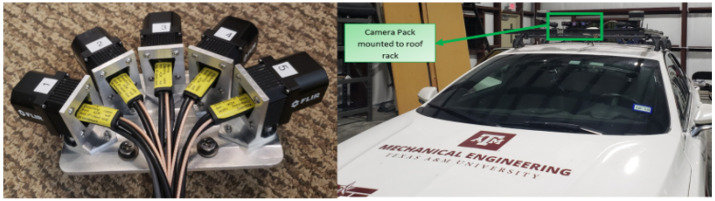
Thermal camera assembly. Each of the five thermal cameras used for the image stitching are shown with the numbers 1–5.

After iterating over the design using a 3D printer, the final design of the mount was fabricated in aluminum. The final assembled thermal camera system and its mounting location (roof of the car) are shown in Figure 4.

**Figure 5:**
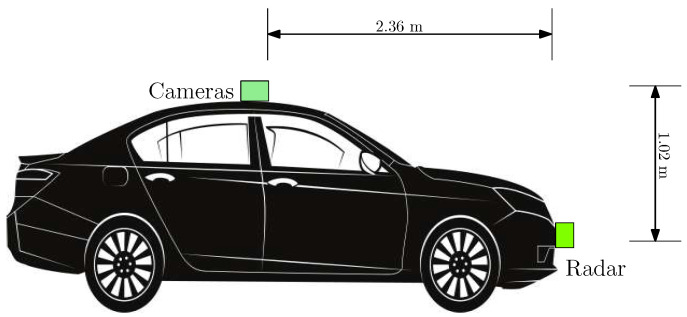
Relative position of the thermal cameras and radar. Reprinted with permission from earlier conference paper Ref. [11]. ©2021, SAE International.

The measured relative distance between the camera and radar is shown in Figure 5. These distances were used for translating radar sensor measurements into the camera coordinate frame. The camera and radar were along the same geometric center line of the vehicle, facing the longitudinal direction of the car.

In addition to these thermal cameras, an RGB camera was also mounted to the dashboard inside the vehicle to serve only as a qualitative reference. The data from this RGB camera was not used as input to the object detection or tracking algorithms for this paper. A GPS/IMU module from Xsens was used to obtain vehicle acceleration and heading necessary for sensor fusion, while the vehicle speed was recorded from the onboard CAN bus.

### 2.2. Software

We used an open-source platform, ROS (Robot Operating System) [19], to serve as the middle-ware for all the sensors and to record the data. All the sensors, CAN bus, and IMU information were recorded at a minimum of 20 Hz sampling rate.

**Figure 6:**
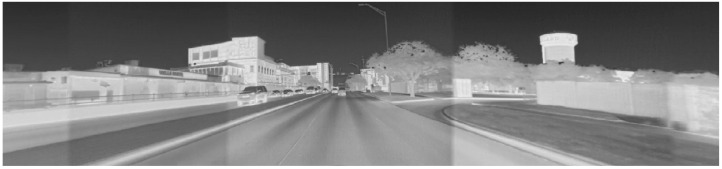
A screenshot of a stitched video feed from the thermal cameras.

As mentioned earlier, the video streams from each camera were digitally stitched in real-time to generate a panoramic view in the LWIR spectrum with a net FOV of 190 degrees. This was accomplished with OpenCV and Python using perspective transformations. An initial guess of the transformation matrix was obtained using the geometry of the designed mount and was later fine-tuned manually to obtain a smoothly stitched image. Such a panoramic field of perception is more conducive to tracking objects for a longer duration during self-driving. A sample screenshot of the stitched video feed is provided in Figure 6. All data were collected in or around the City of College Station, TX, USA.

Once we obtained this panoramic thermal image, objects in the scene need to be identified. We used a well-known state-of-the-art convolutional neural network-based object detector, YOLOv3 [20], for this purpose.

We could not simply use a pre-trained YOLO network since the input layer for the network expects images that have a nearly square (1:1) aspect ratio. As we can see from Figure 6, the output of the thermal camera system has an aspect ratio of nearly (5:1). Therefore, the network had to be retrained for our purpose. We chose to focus on the ’vehicle’ class since it reflects the primary mode of transportation in College Station, Texas, and allows us to obtain sufficient data for testing.

**Figure 7:**
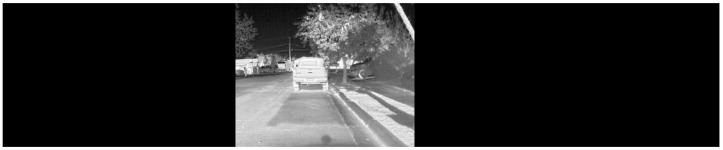
Random padding added to FLIR dataset images for training.

For training, we used an annotated thermal imaging dataset provided by FLIR [21]. The dataset provided by FLIR contains images of size 512×640 pixels, yielding a nearly (1:1) aspect ratio. In order to reflect the panoramic thermal camera system output, each image in the FLIR dataset was padded on either side to generate a (5:1) aspect ratio dataset. The size of the padding on each side was randomized while keeping the total padding width constant so that the image from the annotated dataset was not always at the center (as shown in Figure 7).

These padded images were then used for training the object detector after making the following modifications to the configuration of the YOLO network:

- The width and height parameters of the network were changed to 1280 and 256, respectively. This would enable better detection of the 5:1 aspect ratio of the stitched video streams from the thermal camera assembly.
- The number of channels was changed from 3 (Red, Green, Blue) to 1 since the thermal cameras only output gray scale pixel information.
- The saturation and hue parameters were eliminated since these are only applicable for visible spectrum data.

**Figure 8:**
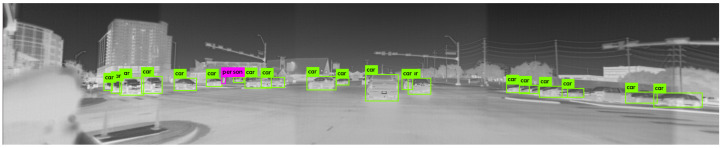
An example of the output of the object detector running on the thermal images.

The network was trained for 12,000 batches, after which it yielded satisfactory performance and was able to reliably detect cars and pedestrians both during day and at night. A sample output of the custom-trained YOLOv3 object detector is shown in Figure 8. The image was captured at night at a busy intersection, and we can see that most of the vehicles are correctly identified with colored bounding boxes.

The position of the centers of these bounding boxes and their width/height are considered measurement outputs from the thermal camera system. Likewise, the radar provides the relative position and relative velocity of the object detected. These sensor measurements are then fused to create continuous tracks, as described in the next section.

## 3. Methodology: Sensor Fusion Multiple Object Tracking

Once the sensor measurements are received, they need to be processed to remove false measurements (‘clutter’) and identify targets of interest. Once targets are established, they need to be tracked as it is important to have an estimate of the future trajectory of all road users for automated driving. For every new measurement received, a decision has to be made whether the measurement corresponds to any of the vehicles being tracked, if it necessitates the creation of a new trajectory or if it should be discarded as clutter. This process of creating new trajectories (tracks) from sensor measurements, updating the existing tracks and pruning out unlikely tracks is usually referred to as data association.

A variety of approaches for data association for multiple target tracking have been published in the literature dating back to the late 1970s [22,23,24]. Many of the conventional approaches can be broadly separated into two classes: Single Frame vs. Multi Frame data association, depending on whether the measurement-to-track association is made on a frame-by-frame basis or if a history of ‘likely assignments’ is stored for a complete decision to be made later [25]. The simplest of the single-frame methods is the Nearest Neighbor association, where the sensor measurement closest to the track is associated with it, and the rest are discarded as clutter. An optimal version of this algorithm, referred to as the Global Nearest Neighbor association, uses the Kuhn–Munkres/Hungarian algorithm [26]. Alternatively, Joint Probabilistic Data Association (JPDA), first proposed by [27], uses a Bayesian approach to data association, effectively utilizing a weighted sum of all measurements in the neighborhood of a track. Multiple Hypothesis Tracking (MHT) is an example of the multi-frame association [28], and newer approaches also exist based on Random Set theory [29] and Particle Hypothesis Density [30]. A review of data fusion strategies is also available in [29].

**Figure 9:**
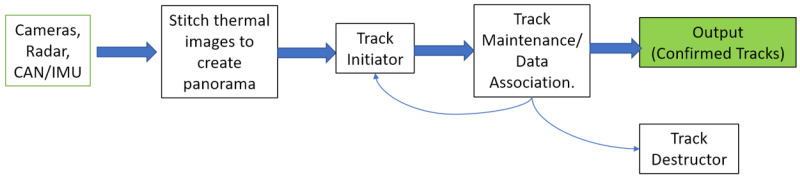
Overview of the object tracking algorithm.

In this work, we focused on JPDA and MHT since they are the most commonly used data association techniques and were designed specifically for applications involving radars. We hope to evaluate other techniques in the future. Both methods were implemented and evaluated. The overall architecture of the sensor fusion algorithm is shown below in Figure 9. The JPDA and MHT alternatives largely differ in the Track Maintenance block.

### 3.1. Model Assumptions

We represent object tracks as state vectors in the vehicle frame of reference with the thermal camera as the origin. Since the YOLOv3 algorithm yields the size of the bounding boxes as well as the center point of the boxes, we use the following state vector for the camera frame:

(1) $${\vec{X}}_{cam}={\left[P_{y},P_{z},\dot{P_{y}},\dot{P_{z}},W,H,\dot{W},\dot{H}\right]}^{T},$$

where $P_{y}$ and $P_{z}$ represent the coordinates of the center of each bounding box in the camera coordinate frame (in pixels) with $\dot{P_{y}}$ and $\dot{P_{z}}$ as their derivatives. We use a right-hand coordinate system with the longitudinal direction of travel of the vehicle selected as the +x-direction and the +z-direction pointing upwards from the surface of the road. $W,H,\dot{W}$, and $\dot{H}$ denote the width and height of the bounding boxes, along with their derivatives. For the vehicle dynamics model, we implement an extended Kalman Filter from our research group’s previous work on truck platooning [31].

### 3.2. Track Initiation

For track initiation, we compare data from two consecutive frames for each sensor. If any measurement in the second frame falls within a small neighborhood (5 pixels for the camera and 30 cm for the radar) of measurement in the first frame, then we initiate a track. These bounds on the neighborhoods are reasonable since the sensors collect data at 20 Hz, with only 50 milliseconds between two consecutive measurement frames. Three-frame track initiation and Markov chain-based initiators are also available in the literature [32], but we were able to achieve reasonable performance with this method for our application.

### 3.3. Validation Gates and Track Maintenance

**Figure 10:**
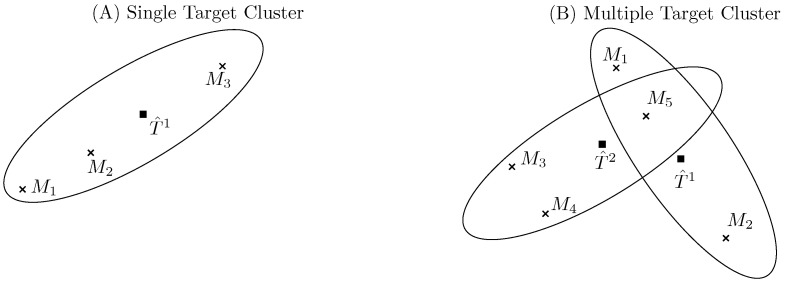
Examples of validation gates for single and multiple target clusters.

Once a set of tracks have been initiated, the camera and radar trackers group the incoming sensor measurements into clusters. This is performed using ellipsoidal validation gates, which are picked so as to maximize the probability that the true measurement from the track is within this region while minimizing the volume of the region. If two tracks are spatially close together, their validation gates may overlap, as shown in the right sub-figure of Figure 10. In the figure, ${\hat{T}}^{1}$ and ${\hat{T}}^{2}$ are the current estimates of the target states and $M_{i}$, for i=1,2,…,5 are newly received sensor measurements. We can see that in this example, $M_{5}$ lies in the validation regions of both the targets of interest, so ${\hat{T}}^{1}$ and ${\hat{T}}^{2}$ would be clustered together.

A sensor measurement at time *k* is said to be within the validation region for a target if the Mahalanobis distance between the predicted target position and the measurement is less than some threshold value, as represented in the following equation:

(2) $${\left[s_{k}-{\hat{s}}_{k|k-1}\right]}^{T}S_{k}^{-1}\left[s_{k}-{\hat{s}}_{k|k-1}\right]\leq g^{2},$$

where $s_{k}$ is the measured state vector from the sensor at time *k*, ${\hat{s}}_{k|k-1}$ is the predicted state obtained from the Kalman estimator based on information up to time k−1, and $P_{k}$ is the covariance of the innovation term in a standard Kalman Filter formulation [25]. The threshold *g* is representative of the volume of the validation gates.

The radar measurements were observed to contain a large amount of ‘clutter’. For example, the radar often picks up trees and curbs on the sidewalk or any uneven portions of the road as obstacles due to its positioning close to the surface of the road. Given the low resolution of the radar, it does not offer any distinction between such clutter and targets of interest, so we rely on the data association algorithm to maintain tracks.

#### 3.3.1. Joint Probabilistic Data Association

While the Nearest Neighbor approach (simply selecting the nearest sensor return at the next time step) works well for single object tracking, it performs poorly when clusters of objects are present in the scene. Moreover, it was observed that the Delphi ESR radar installed on the car often returns multiple measurements for the same object. For example, a single vehicle in front could return two or three “blips” on the radar. This violates the one-to-one correspondence assumption of the Nearest Neighbor tracker. The Joint Probability Data Association (JPDA) tracker presents a Bayesian approach to tracking, which takes into account the possibility of many-to-one measurement to target correspondence.

The JPDA algorithm requires the generation of all possible ‘feasibility’ event matrices [32], which is a combinatorial problem. For example, in the multiple target scenario presented in Figure 10 with two targets and five measurements, the total number of feasibility matrices is 31 (see Table 1 below, which is an excerpt from [33]).

Generating these event matrices and calculating conditional probabilities for each can be time-consuming, especially if there are a large number of measurements associated with each cluster. To overcome this, Zhou and Bose [34] proposed a formulation that circumvents the need to generate event matrices, provided the density of tje number of targets per cluster is low. In our dataset, we observed that the majority of clusters have three or less targets per cluster, so the faster JPDA algorithm from [34] was implemented.

#### 3.3.2. Multiple Hypothesis Tracking

While a brief summary of our implementation is provided below, our previous work [11] contains a more detailed explanation.

MHT is one of the widely used algorithms for multiple target tracking. The key idea behind MHT is to delay the data association process until more information is obtained. To achieve this, separate tracks are maintained for each possible data association. At every time step, the predicted track position from a Kalman Filter is used to establish the validation gates for each track. For any new observations that lie inside the validation gates of a track, a new track is generated. A track is also kept to represent a missed detection case. A hypothesis is the collection of tracks that do not share observations at any time step.

Following the work by Sitlller [35], we used the log-likelihood ratio between the target hypothesis and the null hypothesis as the track score. The target hypothesis assumes that the sequence of observations comes from the same target, and the null hypothesis assumes that the sequence of observations comes from the background.

After getting the score for each track, we attempt to determine the most likely hypothesis (or Global Hypothesis) and then eliminate unlikely tracks by using several pruning techniques. The score of a hypothesis is the sum of the scores of all the tracks in the hypothesis. In order to find the global hypothesis, we first generate a graph where each node represents a track with a weight equal to the track score. An edge is added between two nodes if they belong to the same cluster, i.e., they share an observation. Next, we calculate the Maximum Weighted Independent Set (MWIS) to find the global hypothesis.

MHT is capable of thoroughly exploring the large solution space of data association problems but has traditionally been limited by the exponential growth in the number of hypotheses. To handle these challenges, several pruning techniques are used, such as *N*-scan pruning or propagating only the *M*-best hypotheses. Even after using these pruning techniques, we discovered that it is difficult to achieve real-time tracking in busy urban traffic. One of the most computationally expensive steps in the MHT algorithm is the calculation of the global hypothesis, and since MWIS is an NP-hard problem, it becomes increasingly time-consuming as the number of tracks grows with the rise in traffic and measurements.

If the tracks are over-pruned then the algorithm may no longer be reliable. We have introduced a few modifications in track initiation and update steps to curb the hypothesis’s growth without sacrificing the performance. First, if a track has at least one observation inside its validation gates, we will not add a no-data association track to the list as it covers the case of a missing observation. Second, if an observation lies inside the validation gates of any existing track, we will not generate a new track associated with that observation. We will also limit the maximum number of tracks in the global hypothesis as we can only have a finite number of vehicles to track at a time on the road.

We also implemented a strategy to address the challenge of multiple detections generated by radar for one object. Conventional MHT does not address this problem and assumes that each object can only generate one detection. We have addressed this issue by using the bounding box information from the thermal camera system. In the graph generated to find the global hypothesis, we add an edge between any two tracks that share a common bounding box. By including this modification, the global hypothesis will always have only one track corresponding to each object tracked. Please refer to our previous conference paper [11] for more details on our MHT algorithm implementation.

### 3.4. Track Destruction

When objects permanently move outside the field of view of the radar and imaging systems, their corresponding tracks need to be destroyed for computational efficiency. We achieve this by maintaining a time-to-death counter for each tracked object. The counter is dropped by 1 for every time step when none of the new measurements could be associated with an existing track and is reset if a measurement is successfully assigned to that track in the future. If the counter drops below a threshold, the track is deleted.

## 4. Results and Validation

**Figure 11:**
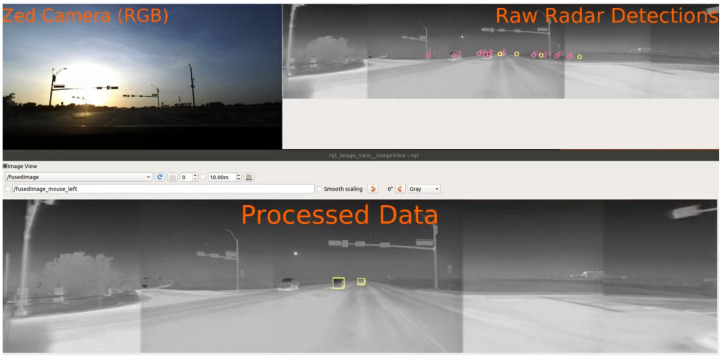
A screenshot showing the object tracking algorithm running under poor visibility conditions. The pink and yellow circles on the top right show the confirmed detections and clutter respectively from the radar. The yellow boxes in the bottom show the final vehicle tracks after sensor fusion.

The proposed architecture is able to work in near real-time with the thermal cameras running at 20 fps. A screenshot of the algorithm tracking the vehicle ahead in spite of glare from direct sunlight is shown in Figure 11. The top left insert shows the visible spectrum camera output for reference. The top right insert contains raw, noisy radar measurements projected onto the camera frame, and the bottom insert shows the final tracked objects.

A video file is available for playback at the https://youtu.be/J4EtQSkiTvE (accessed on 14 June 2022).

The object tracker was observed to perform well with vehicles that are traveling in the same direction as the ego vehicle but is unable to track the oncoming vehicles reliably. This is primarily due to the way the heading angles of objects are currently initialized within the radar tracker.

### Quantifying the Performance Using a Digital Twin

Now, we need to quantify the algorithm’s tracking performance. In order to do this, we need the ‘ground truth’ of the vehicle positions in the scene. Ideally, this would mean obtaining the pose of each vehicle using an IMU and GPS mounted to each vehicle on the road. This is not a practical solution. Therefore, we first explored utilizing a high-resolution lidar sensor as a source for the ground truth. We mounted an Ouster OS-1, a 128-channel high-resolution lidar onto the roof of the vehicle and examined the lidar data. The goal was to annotate the lidar point-cloud to identify the voxels that correspond to vehicles being tracked. These voxels can then be projected onto the thermal camera frame and compared with the output of the vehicle tracker.

**Figure 12:**
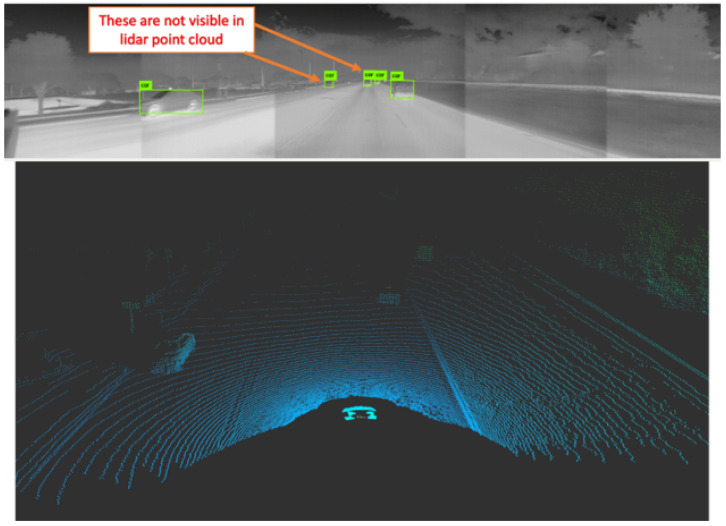
The thermal camera system was able to pick up far-away vehicles not visible on the lidar. The green boxes represent the output of the YOLO object detector picking up cars from the thermal image.

Unfortunately, we noticed that the lidar had a lower detection range compared to that of the thermal system. That is, the thermal camera system was able to track vehicles that were further away. These far-away vehicles had no corresponding voxels in the point cloud. An example of this scenario is shown in Figure 12.

**Figure 13:**
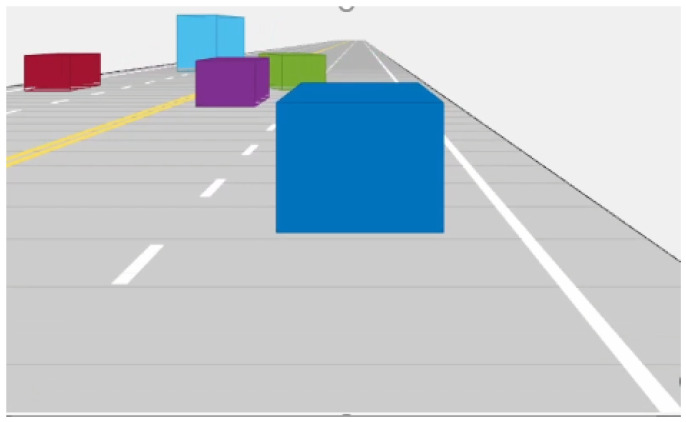
A screenshot of the scenario designed in Driving Scenario Designer. Each color shows a different vehicle.

Therefore, validation had to be performed in a simulated setting. We chose MATLAB in combination with Unreal Engine 4 to accomplish this task. First, a scenario was designed in MATLAB using the Driving Scenario Designer, which allows us to set the trajectories of the ego vehicle as well as any surrounding vehicles. For this work, we added four vehicles to the scene, two traveling in the direction of the ego vehicle and two in the opposite direction. These vehicles perform sinusoidal (double lane change) maneuvers while traveling at 25 m/s. A screenshot of the scenario designed is shown in Figure 13.

**Figure 14:**
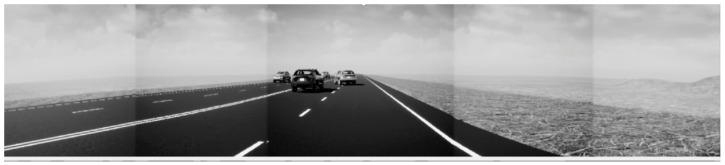
A screenshot showing a virtually generated panorama, as seen through simulated cameras, from the ego vehicle. Image is in gray-scale to reflect the output type of thermal images.

Then, we use the Unreal Engine plugin to generate a realistic scene from these vehicle trajectories. We also incorporated five camera models using in-built functions in MATLAB’s Automated Driving Toolbox. Each of the cameras was set to have a field of view of 50deg and the same camera intrinsic parameters as that of the FLIR camera. This allows us to use the same stitching algorithm to virtually generate a panoramic image, as shown in Figure 14. Please note that Unreal Engine was the closest approximation we could obtain in order to generate a digital twin. In order to truly simulate a thermal camera, one would have to incorporate thermodynamic principles (conduction, convection, radiation) into the visualization software. To the authors’ knowledge, at the time of publication, there was no such comprehensive photo-realistic visualization software for thermal cameras. That said, the authors have undertaken due diligence to maintain the accuracy of the digital twin as much as possible. The camera’s intrinsic parameters were programmed into the simulation to match that of the real thermal cameras (obtained from [36]). The relative position of the thermal camera and radar was also measured on the real vehicle and incorporated into the simulation.

Since the characteristics of the Delphi ESR radar have been experimentally measured by other researchers [37], we were able to readily use this information for tuning the parameters of the “Simulation 3D Probabilistic Radar” block in MATLAB to simulate the forward-facing radar. Since the pose of the ego vehicle is known, we were able to use this to generate the IMU and vehicle CAN bus data. All these simulated sensor data streams were published as ROS topics in the same message format as that recorded from the real vehicle. In this way, we were able to generate a complete digital twin for validation.

Finally, we use py-motmetrics library (https://github.com/cheind/py-motmetrics (accessed on 14 June 2022))to calculate identification precision and recall scores of the tracking algorithm. These calculations are performed in the camera frame of reference. Since we know the ‘ground truth’ in these simulated scenarios, we can project the location of each vehicle being tracked into the camera frame and compare with the output of the tracking algorithm. Table 2 shows the scores obtained for the MHT and JPDA algorithms.

These values are in the same neighborhood as those from open-source benchmarks (such as from py-motmetrics repository), though there is some need for improvement in the IDR (recall) performance. This is directly related to the performance of the object detection network (YOLOv3) that we had trained for this work, which can be improved by using a larger training dataset.

## 5. Summary

If vehicles are to be fully autonomous, they need to perceive and track objects in the environment even under non-ideal conditions. There are certain scenarios in which the currently used sensor stack of cameras, lidars, and radars cannot be relied upon. In this paper, we have demonstrated the benefits of using a thermal imaging system to fill this gap and overcome object tracking challenges in poor visibility conditions. We explored single and multiple frame data association techniques and adapted them for this particular application. These algorithms were implemented on a real vehicle and validated using a digital twin with virtual sensors. The collected thermal, radar, lidar, and vehicle data have been made available online. In the future, we hope to improve object detection performance and expand testing to demonstrate the efficacy of the system in heavy fog and snow using artificial fog machines/tunnels and by testing in different geographical locations. As part of future work, we would also like to evaluate other data association techniques.

## Figures and Tables

**Table 1:** Total Number of Feasibility Matrices for a Given Number of Targets per Cluster, adapted from Ref. [33].

Measurements per Cluster			Number of Targets	
	1	2	5	10
1	2	3	6	11
2	3	7	31	111
5	6	31	1546	63,591
10	11	111	63,591	2.34 × 10^8^

**Table 2:** Comparison of precision and recall scores from validation.

	Identification Recall (IDR)	Identification Precision (IDP)
JPDA	44.7%	38.2%
MHT	68.0%	49.0%

## Data Availability

The collected thermal camera, radar, lidar, vehicle CAN, and IMU data are available as ROS bag files from https://doi.org/10.15787/VTT1/B3VKEA (accessed on 14 June 2022).
